# Which Fibers of the Medial Collateral Ligament (MCL) Should Be Released in the Pie Crust Technique Applied During Knee Arthroscopy: Superficial MCL or Deep MCL?

**DOI:** 10.7759/cureus.20597

**Published:** 2021-12-22

**Authors:** Gökhun Arıcan, Niyazi Ercan, Melih Elçi, Özgür Şahin, Bahadır Alemdaroğlu

**Affiliations:** 1 Department of Orthopedics and Traumatology, Yuksek Ihtısas University, Medical Park Ankara Hospital, Ankara, TUR; 2 Department of Orthopedics and Traumatology, Ankara Training and Research Hospital, Ankara, TUR

**Keywords:** knee arthroscopy, meniscal tear, release, medial collateral ligament, pie crust

## Abstract

Background

Knee arthroscopy is the most common surgery performed to treat meniscal injuries. The pie crust (PC) technique is applied during knee arthroscopy to increase joint vision of the medial femorotibial compartment and reduce the risk of iatrogenic damage. Medial collateral ligament (MCL) release is applied in the PC technique. Currently, there are no studies directly comparing the release of the superficial MCL (sMCL) or deep MCL (dMCL) when applied during the PC technique. In this study, we compared the clinical and functional results of the release of the deep and proximal tibial attachment of the superficial fibers of the MCL.

Methodology

We evaluated the results of 67 (27 women and 40 men) patients who underwent the PC technique during knee arthroscopy due to a medial meniscal tear. The patients who underwent the PC technique were divided into two groups according to the release of the deep and superficial fibers of the MCL. All patients were evaluated for pain, functional capacity, and laxity using the Knee Injury and Osteoarthritis Outcome Score (KOOS) and the Oxford Knee scores. All patients were evaluated with radiographic examinations such as valgus laxity angle and medial tibiofemoral compartment opening height.

Results

The KOOS and Oxford Knee Scores in both groups showed a statistically significant increase at 12 months postoperatively compared with the preoperative values (p = 0.005, 0.002, 0.002, and 0.01, respectively). No statistically significant difference was found between the groups (p > 0.05). When the valgus laxity angle before the PC technique was compared with the 12-month result after the procedure, no statistically significant difference was noted (p > 0.05). There was no evidence of complications such as chondral injury and saphenous nerve or vein injury among patients in either group.

Conclusions

In this study, we did not observe laxity in the long-term follow-up of the groups in which the superficial or deep fibers of the MCL were released. In our view, the PC technique has similar effects on surgical outcomes regardless of sMCL and dMCL release techniques.

## Introduction

Knee arthroscopy is the most common surgery performed to treat meniscal injuries. Complete visualization of intra-articular anatomy is required for the evaluation and surgical intervention of knee pathology [1]. However, in patients with tight tibiofemoral joint space, access to the posteromedial region is challenging.

Instruments used to repair meniscal tears can cause cartilage damage, especially in patients with tight tibiofemoral joint space. In a study evaluating 3,714 patients, the prevalence of iatrogenic cartilage damage during arthroscopy was found to be 2% [2]. Because cartilage tissue is aneural, lymphatic, and avascular, iatrogenic damage can cause articular cartilage degeneration and osteoarthritis [3]. Recent studies have suggested partially releasing the medial collateral ligament (MCL) to increase the medial tibiofemoral joint to reduce the risk of chondral injury.

The “pie crust” (PC) technique is the most commonly performed minimally invasive procedure among MCL release techniques. In the PC technique described by Agneskirchner and Lobenhoffer in 2004, the posterior and proximal fibers of the superficial MCL (sMCL) and posterior oblique ligament were released percutaneously [4]. However, some recent studies have advocated the use of the deep MCL (dMCL) release technique. In a review, Campos et al. stated that there is no consensus on which fibers of the MCL (sMCL, posterior oblique, dMCL) should be released [5].

In this study, we compared the clinical and functional results of releasing the deep and superficial fibers of the MCL. We hypothesized that loosening of the superficial or deep fibers of the MCL was not functionally, radiologically, and clinically different in the PC technique.

## Materials and methods

Approval for this retrospective study was granted by the Institutional Ethics Review Board of Health Sciences University Ankara Training and Research Hospital with the registration number E-93471371-514.10. This study was conducted among 67 (27 female and 40 male) patients who underwent the PC technique during knee arthroscopy due to a medial meniscal tear in Ankara Training and Research Hospital between 2019 and 2021. The inclusion criteria included patients under the age of 55, without cartilage damage (Outerbridge grade 1 and 2), knee arthroscopy for medial meniscus tears, patients who underwent the PC technique. Exclusion criteria included MCL injuries, coronal malalignment with more than 5° of varus and valgus, and grade 3 and 4 cartilage lesion according to the Outerbridge classification. Patients who underwent the PC technique were divided into two groups according to the release of the deep and superficial fibers of the MCL.

Surgical technique

**Figure 1:**
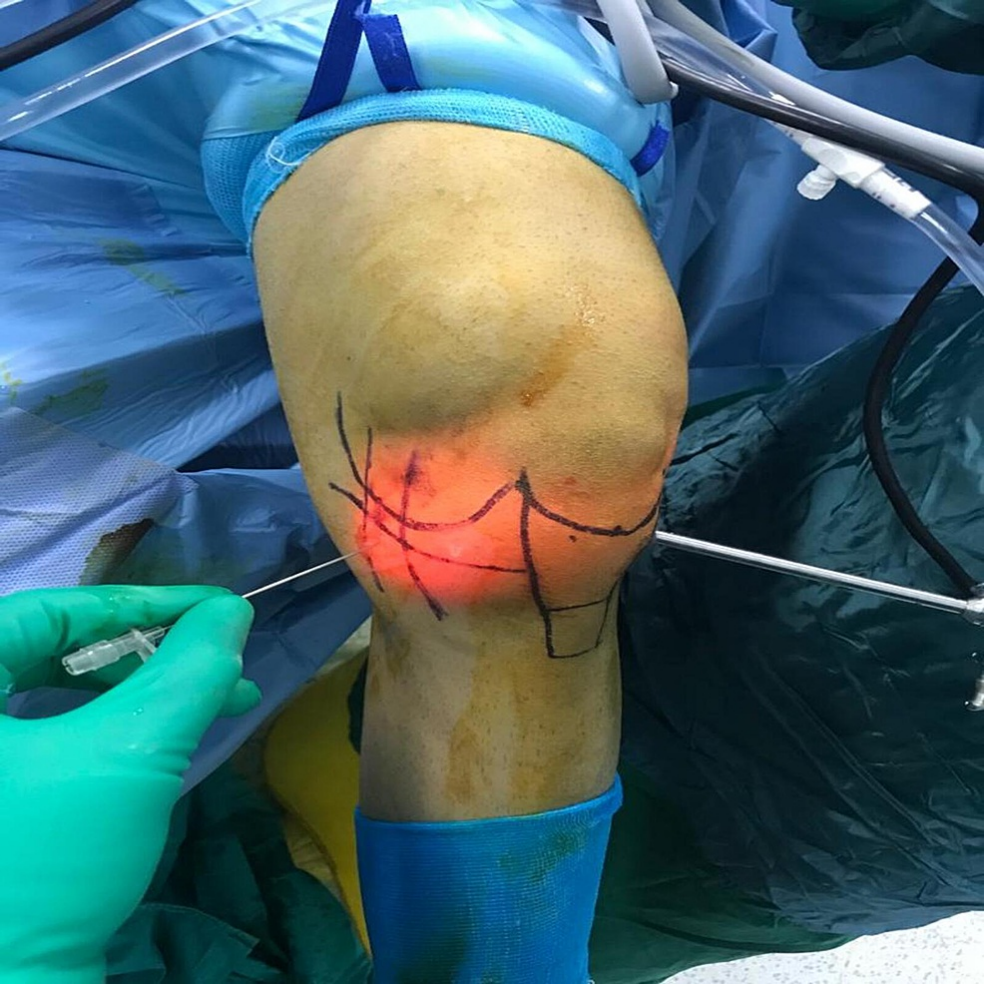
Percutaneous pie crust application using an 18-gauge needle.

The medial femorotibial joint space was evaluated using a probe (5 mm; Stryker, Kalamazoo, Michigan, US). Intraoperative radiographic knee anteroposterior radiographs were obtained for all patients, and a PC was applied for patients who were found to have a femorotibial joint space of less than 5 mm. The percutaneous release was applied for all patients in both groups using an 18-gauge needle under 150 N valgus force using a dynamometer (MicroFET2, Hoggan Scientific, Salt Lake City, Utah, USA) (Figure 1).

No specificity has been determined for superficial or deep fiber release of the MCL. Because there is no consensus in the literature, the difference in preference between surgical techniques was evaluated in this study. The technique familiar to the surgeon was preferred for release. The only criterion defined was narrow femorotibial joint space.

**Figure 2:**
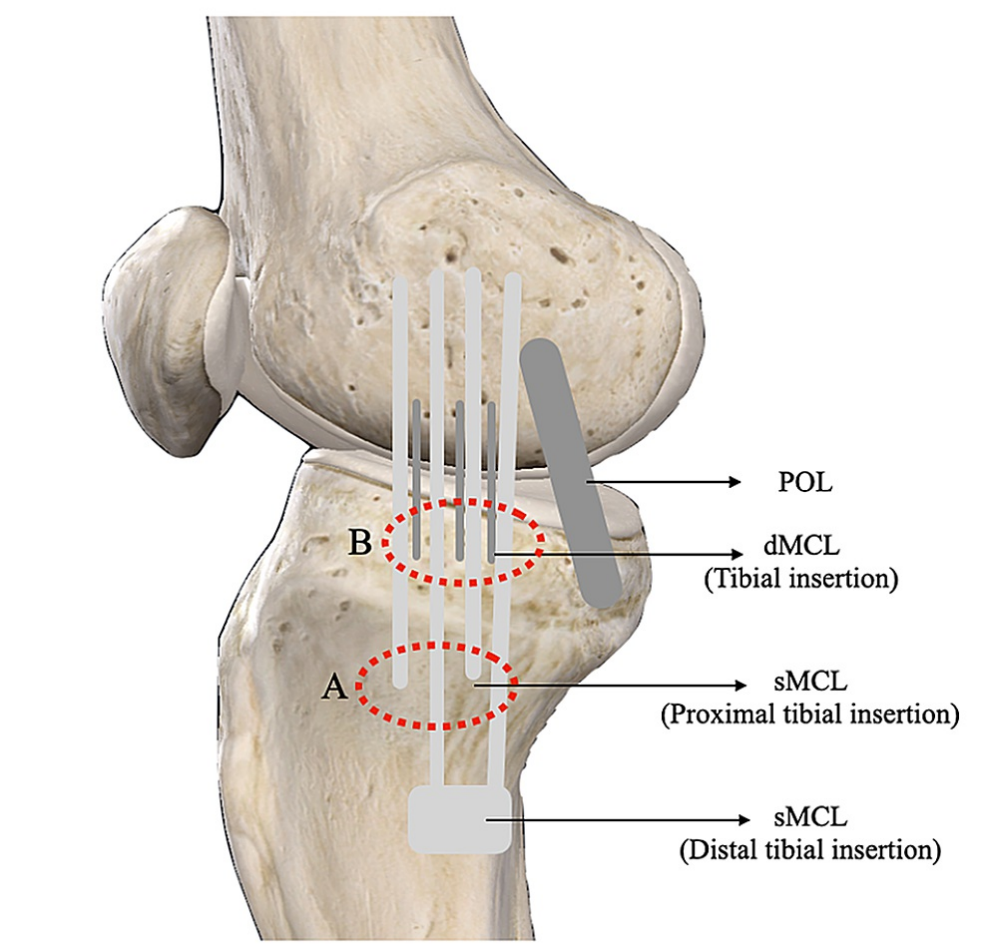
Release points of the deep and superficial fibers of the MCL. A: sMCL release. B: dMCL release. POL: posterior oblique ligament; dMCL: deep medial collateral ligament; sMCL: Superficial medial collateral ligament

The landmark was determined using the anatomy of the sMCL and dMCL, as described by LaPrade et al. [6]. The joint line, POL, sMCL distal tibia insertion, and pes anserinus insertion were marked in all patients. The proximal tibia attachment point of the sMCL was detected between the distal attachment point and the meniscocapsular ligament. Control was achieved using transillumination from the anterolateral portal, and the sMCL was released following the outside-in technique (Figure 2A). The dMCL was controlled by anterolateral transillumination from the tibial capsuloligamentous junction and released from the bone attachment area following the outside-in technique. For dMCL release, first, the 18-gauge needle was observed inside the joint under anteromedial scope control. At the posterior third of the medial meniscus, the dMCL and the meniscocapsular ligament were released posterior to anterior by repeated perforations (Figure 2B).

**Figure 3:**
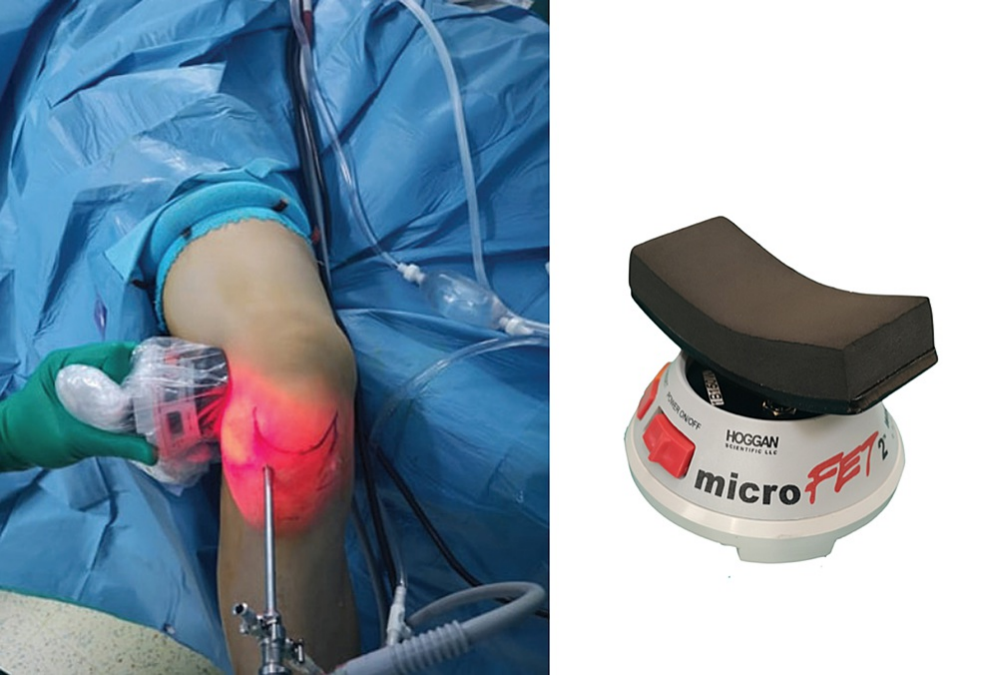
MicroFET2 device used for applying valgus force.

While 150 N valgus force was applied in all patients using a dynamometer (MicroFET2, Hoggan Scientific, Salt Lake City, Utah, US), dMCL or sMCL was released by making several deep skin punctures using an 18-gauge needle. Frequently, the release was confirmed by a popping sound associated with an acute increase in medial joint space (Figure 3).

Assessment methods

Due to the risk of iatrogenic injury, we routinely take knee radiographs for every patient undergoing MCL release. Therefore, all patients were evaluated with radiographic examinations at one, six, and twelve months after surgery. Anteroposterior, 0°, and 30° valgus stress radiographs were obtained and measured, following the methodology described by Arıcan et al. [3]. First, for the measurements, on the X-ray, a line was drawn to connect the subchondral bone of the medial and lateral tibial condyle. From this line, a perpendicular line was drawn to the most distal point of the medial femoral condyle. The length of this vertical line was measured accurately to 0.1 mm and recorded as the joint space width. This measurement is defined as the medial femorotibial compartment opening height [7,8].

Except for radiographic measurements, all patients were evaluated for pain, functional capacity, and laxity using the Knee Injury and Osteoarthritis Outcome Score (KOOS) and Oxford Knee scores before and after surgery.

For rehabilitation, during the postoperative follow-ups, standard rehabilitation protocol was applied without a brace. Patients were allowed to bear weight as tolerated, and exercises were provided to the patients to improve the range of motion and strengthen the quadriceps muscles.

Statistical analysis

Statistical analysis was performed with SPSS version 22.0 software (IBM Corp., Armonk, NY, USA). We examined all variables using visual (histogram and probability graphs) and the Kolmogorov-Smirnov test to assess the normal distribution, as well as applied tests suitable for distribution. Here, we present normally distributed data as mean with standard deviation or median-maximum. We present non-normally distributed data as median with minimum-maximum. We used the Pearson chi-square test for comparing categorical variables such as sex and other characteristics presented as percentages. The independent t-test was used for comparing normally distributed means of two independent groups. For comparison of more than two non-normally distributed groups of time-dependent measurements, the Friedman test was used. The analysis of variance (ANOVA) test was used for comparing more than two normally distributed time-dependent group measurements. We used post hoc tests (Tukey) after ANOVA and (Bonferroni) Friedman’s test for pairwise comparisons. A p-value of <0.05 was considered statistically significant.

## Results

A total of 67 patients who met the inclusion criteria were included in this study. Overall, there were 40 males and 27 females, with a mean age of 48 ± 3 years. Meniscus repair was performed in all patients following the diagnosis of a medial meniscus tear. The demographic data and knee outcomes of the patients included in this study are presented in Table 1.

**Table 1:** Demographic data and knee outcomes of patients. *Row percentage; **Pearson chi-square test; ***independent t-test sMCL: superficial medial collateral ligament; dMCL: deep medial collateral ligament; BMI: body mass index

Characteristic	sMCL release group (n = 34)	dMCL release group (n = 33)	P-value
Age (years)	47 ± 5	49 ± 4	0.83*
Gender			
Female	15 (55.5%)	12 (44.5%)	0.51**
Male	19 (47.5%)	21 (52.5%)	
BMI (kg/m^2^)	27 ± 4	24 ± 3	0.32***
Side			
Right	25 (73.5%)	17 (51.5%)	0.06**
Left	9 (26.5%)	16 (48.5%)	
Follow-up (months)	15 ± 3	16 ± 2	0.62***
Complication			
Iatrogenic chondral injury	-	-	
Iatrogenic saphenous nerve injury	-	-	
Iatrogenic saphenous vein injury	-	-	

The KOOS and Oxford Knee scores in both groups showed a statistically significant increase at 12 months postoperatively compared with the preoperative values using the ANOVA test (p = 0.005, 0.002, 0.002, and 0.01, respectively). No statistically significant difference was found between the groups one year postoperatively (p = 0.72 and 0.54, respectively) (Table 2).

**Table 2:** The value of clinical scores ^a^The KOOS (%); ^b^Oxford score; *ANOVA test; **pairwise comparison with Tukey post hoc test. KOOS: Knee Injury and Osteoarthritis Outcome Scores; Oxford: Oxford Knee score; sMCL: superficial medial collateral ligament; dMCL: deep medial collateral ligament; ANOVA: analysis of variance

Clinical scores	Groups	Time				P-value at pre and postoperative one year within groups	P-value at pre and postoperative one year between groups
		Preoperative	Postoperative (One month)	Postoperative (Six months)	Postoperative (One year)		
KOOS^a^	sMCL release group	65 ± 6	54 ± 5	70 ± 12	81 ± 6	0.005*	0.72**
	dMCL release group	60 ± 8	65 ± 5	77 ± 6	82 ± 4	0.002*	
Oxford^b^	sMCL release group	38 ± 4	42 ± 3	51 ± 2	54 ± 2	0.002*	0.54**
	dMCL Release Group	36 ± 3	38 ± 2	47 ± 3	53 ± 2	0.01*	

No statistically significant difference was found when the KOOS scoring system, which evaluated symptoms, quality of life, sports activity, and functional measurements, was compared between the groups (p < 0.05) (Table 3).

**Table 3:** KOOS scoring system results regarding pain and functionality. *Independent t-test was used. KOOS: Knee Injury and Osteoarthritis Outcome Score; QOL: quality of life; ADL: activities of daily living; sMCL: superficial medial collateral ligament; dMCL: deep medial collateral ligament

KOOS	sMCL release group	dMCL release group	P-value*
Symptoms	88.2 (78-90)	86.5 (78-88)	0.53
QOL	84.6 (80-94)	83.5 (74-89)	0.63
Sport/recreation	72.4 (68-85)	74.4 (70-81)	0.75
ADL	80.2 (75-90)	85.8 (77-84)	0.58
Total score of KOOS	81 (75-87)	82 (78-86)	0.85

The results of the radiological measurements (medial femorotibial compartment opening height and valgus laxity angle) are presented in Table 4 for both groups. On pairwise comparisons of the medial femorotibial compartment opening height measurements before the PC technique and the measurements at one and six months after the PC technique, it was found to be statistically significant (p = 0.036). However, there was no significant difference between the preoperative and 12-month postoperative medial femorotibial compartment opening height measurements for the dMCL (p = 0.79) and the sMCL group (p = 0.83). When the valgus laxity angle before the PC technique was compared with the one-year result after the procedure, no statistically significant difference was found for the dMCL release group (p = 0.85) the sMCL group (p = 0.83). There was no statistically significant difference between the groups regarding radiological joint laxity measurements (p = 0.83) and opening height measurements (p = 0.83) (Table 4).

**Table 4:** Radiographic laxity results at follow-ups. *Friedman test (pairwise comparisons used with Bonferroni correction); **angle median comparison between dMCL and sMCL; ***distance median comparison between dMCL and sMCL; ^a^valgus laxity angle (degree); ^b^medial tibiofemoral compartment opening height (mm). Values are presented as median and minimum-maximum. sMCL: superficial medial collateral ligament; dMCL: deep medial collateral ligament

Groups	Measurement	Preoperative	Intraoperative (after pie crust)	Postoperative (One month)	Postoperative (Six months)	Postoperative (One year)	Test P-value *	P-value at pre and postoperative one year	P-value**	P-value***
dMCL release group	Angle^a^	4.7 (4.4-5.6)	8.2 (6.4-9.3)	7.4 (5.9-8.6)	6.2 (5.6-8.2)	4.8 (4.6-5.9)	0.2	0.85	0.83	0.83
	Distance^b^	4.3 (3.5-4.8)	11.2 (8.3-12.5)	9.2 (8.8-10.2)	7.5 (6.8-9.3)	4.8 (4.4-6.7)	0.5	0.79		
sMCL release group	Angle^a^	4.1 (3.3-5.2)	7.5 (6.6-9.1)	6.7 (5.3-8.2)	5.8 (5.2-9.1)	4.3 (4-5.5)	0.1	0.83		
	Distance^b^	4.5 (3.6-4.9)	10.4 (7.9-12.1)	8.4 (7.8-11.2)	6.8 (6.1-9)	4.3 (4-6.3)	0.1	0.83		

There was no evidence of complications such as chondral injury and saphenous nerve or vein injury in patients in either group.

## Discussion

The results of this study showed that in the PC technique the release from the superficial or deep fibers of the medial collateral ligament was not radiologically or functionally different.

Dick et al. reported that the risk of iatrogenic cartilage damage during arthroscopy was 8% and that it was the most common complication [2]. The risk of complications is higher, especially in patients with a tight knee with narrow medial joint space due to anatomical variations. Consequently, narrow medial joint space may lead to inadequate treatment, meniscus injury, cartilage damage, and diagnostic errors due to an insufficient field of view. Agneskirchner and Lobenhoffer in 2004 described MCL release following a needle-assisted inside-out technique to reduce the risk of iatrogenic chondral injury and increase joint visual field [4]. In this technique, they described the percutaneous release of the superficial fibers of the MCL using a needle. In addition, they reported that outside-in and percutaneous application reduces the risk of intra-articular septic complications. In recent years, several studies have described similar methods with minor modifications. These applications are described by the release method (inside-out, outside-in), the fiber released (sMCL, dMCL, or POL), and the equipment used to release (18-gauge needle, banana blade, electrocautery). However, there are no studies directly comparing the results of release from the sMCL or dMCL when applying the PC technique.

The medial stabilization of the knee is provided by the sMCL, dMCL, and POL. Griffith et al. defined sMCL as the primary stabilizer against valgus forces in their anatomical studies and reported that it was responsible for the majority of the resistance against valgus stress in the knee [9]. The superficial fibers of the MCL have two separate insertions, namely, proximal and distal. The proximal part attaches to the posteromedial of the tibial plateau close to the joint, whereas the distal part attaches posterior to the pes anserinus. Robinson et al. reported that one of the important factors in preventing valgus laxity is the distal tibial insertion of the sMCL [10]. Park et al. reported that sMCL release can cause instability [11]. Although several studies have indicated that sMCL is the most important structure in valgus stability, the release of sMCL is widely reported in the literature.

However, Li et al. could not observe laxity in their 20-year follow-up of patients who underwent percutaneous sMCL release [12]. Several studies have reported that the release of POL or sMCL at the joint line does not lead to laxity in short and long-term follow-ups [13,14]. Contrary to these studies, others have shown that releasing the deep fibers of the MCL from the joint level did not cause any laxity [11,15,16]. In our study, in which we obtained similar results, we did not observe laxity in the long-term follow-up of the groups in which the superficial or deep fibers of the MCL were released.

In their cadaver study, Claret-Garcia et al. reported that dMCL release provided a 2.9 mm increase in the medial femorotibial compartment opening height [17]. In another study in which the sMCL was released, the authors reported that the medial femorotibial compartment opening height increased from 2.5 to 5.7 mm [18]. In our study, we observed an increase in the medial femorotibial compartment opening height of 3.8 mm in patients with dMCL release and 4.3 mm in patients with sMCL release. No statistically significant difference was observed when the groups were compared regarding the medial femorotibial compartment opening height. Mihalko et al. applied dMCL release to provide ligament balance in patients who underwent knee prosthesis and determined that the valgus laxity angle was 3.9°, which was 0.9° preoperatively. In another group, Mihalko et al. applied percutaneous release of the sMCL from the proximal tibial plateau attachment site and showed that the valgus laxity angle increased from 1.0° to 3.1° preoperatively [19]. In both studies, they stated that there was no statistically significant difference in the long-term follow-up results of the valgus laxity angle when compared with the preoperative values; however, there was a significant difference in the medium and short term. In our study, in which we obtained similar results, we found that the valgus laxity angle was significantly higher than the preoperative values in the one and six-month evaluations; however, there was no significant difference in the 12-month measurements. In addition, in our study, we found that there was no statistically significant difference in the release of the sMCL or dMCL in terms of valgus laxity angle.

Some studies have reported that patients undergoing MCL release experience short-term pain at the site of the postoperative release [14,18]. Jeon et al. compared patients with and without percutaneous MCL release and showed that patients with MCL release on the first postoperative day had higher visual analog scale scores [20]. Claret et al., in their study on 140 patients, showed that the results of 70 patients who underwent MCL release regarding pain and function at two and six months were more successful than 70 patients who did not undergo MCL release [14]. Leon et al. applied arthroscopic medial release in patients with medial compartment arthritis and showed that the biomechanical and kinematic functions of the patients improved significantly due to the decrease in medial compartment load [21]. Especially in patients with narrow medial compartment, the PC technique can provide a more balanced load distribution with an increase in the medial tibiofemoral joint space and can provide an advantage in terms of pain in the middle term. In our study, no statistical difference was observed in terms of postoperative pain in patients who underwent sMCL or dMCL release. When patients in both groups were compared using the KOOS regarding symptoms, quality of life, sports, and activities of daily living, no statistically significant difference was observed. However, we found that patients in both groups had better pain and functional results after MCL release in the long-term follow-up. More studies with long-term follow-ups are needed to evaluate the effects of MCL release on pain.

Although the percutaneous outside-in technique used in our study has advantages such as being minimally invasive and having a low risk of infection, there is a risk of injury to the saphenous nerve and vein [13]. In a cadaver study by Roussignol et al., a risk of saphenous vein injury during sMCL release was noted at the joint level, while saphenous nerve injury was observed during dMCL release. However, they stated that the risk of injury is rare because the area where the percutaneous release is performed is far from nerve and vascular structures [22]. Despite the theoretical risk of damage to the saphenous nerve and vein during the PC technique, no intraoperative complications were observed in either group of patients.

There is no study in the literature comparing patients with and without braces in the postoperative period after MCL release. Therefore, there is no consensus on the use of braces postoperatively. Jeon et al. used braces for prophylactic reasons due to the risk of MCL damage after PC [20]. In addition, Fakıoğlu et al. reported no evidence for the use of postoperative braces; however, they used braces for ethical reasons [13]. It has been reported that immobilization is not necessary for ligament healing in the postoperative period among patients who underwent percutaneous MCL release, and no complications were observed among patients who did not use a brace [5,15]. In his study, Lyu performed a wider MCL release compared to the classic PC technique for the treatment of medial compartment osteoarthritis and did not use braces during postoperative follow-ups. He did not encounter any complications related to not using braces, including laxity in the short and long term [23]. In our study, all patients were mobilized as much as they could tolerate postoperatively without using braces. No complications due to not using braces were observed in patients in both groups.

The major limitation of our study is that the detection of dMCL and sMCL was done through anatomical landmarks and ultrasonography was not used. Another important limitation is that our study is retrospective. The recovery of MCL could have been evaluated with further investigations such as magnetic resonance imaging. However, we did not evaluate it for ethical and financial reasons. Laxity assessments and medial femorotibial joint height measurements were made by two different specialists, and intra and interobserver assessments were not performed. Therefore, individual measurement errors may have occurred. Moreover, long-term follow-up periods of up to 12 months in terms of diagnosis and follow-up of iatrogenic cartilage lesions are further limitations.

## Conclusions

This is the first study in the literature comparing sMCL and dMCL release during the PC technique. Although different techniques have been used in several studies, there is no consensus on the superior technique. Although we did not detect a statistically significant difference, we observed a significant increase in the femorotibial joint space in patients with dMCL release compared to those with sMCL release. These findings indicate that the medial femorotibial field of view can be increased in patients with dMCL release than in those with sMCL release.

Hence, both methods have similar outcomes. Based on these data, in our view, the PC technique has similar effects on surgical outcomes, regardless of sMCL or dMCL release. In both techniques, compared to the preoperative period, laxity did not develop in long-term follow-ups, the visual field of the medial compartment increased, and pain and functionality improved.
